# Supplementary material for: Gains vs losses in pay-for-performance: Stated preference evidence from a U.S. survey
Source: PLoS One. 2025 Feb 10;20(2):e0318704. doi: 10.1371/journal.pone.0318704 (PMC11809869; doi:10.1371/journal.pone.0318704)
Supplement: S1 Table — (DOCX) [file pone.0318704.s001.docx]

**S1 Table. Incentive Needed to Change Behavior ($): Gain vs. Loss Adjusted for Covariates**

|  | Gain | Loss | Difference: Gain – Loss | p-value |
| --- | --- | --- | --- | --- |
| **Model 1: Main effect** |  |  |  |  |
| Full sample | 2160 | 1180 | 981 | <0.001 |
|  | N = 1263 | N = 1264 | (125) |  |
| **Model 2: Rural stratification** |  |  |  |  |
| Rural | 2328 | 1130 | 1233 | 0.010 |
|  | N = 119 | N = 113 | (479) |  |
| Non-rural | 2137 | 1191 | 952 |  |
|  | N = 1144 | N = 1151 | (127) | <0.001 |
| **Model 3: Experience stratification** |  |  |  |  |
| Experience with incentives | 2311 | 1215 | 1137 | <0.001 |
|  | N = 400 | N = 355 | (224) |  |
| No experience with incentives | 2083 | 1174 | 913 | <0.001 |
|  | N = 863 | N = 909 | (150) |  |
| **Model 4: Training stratification** |  |  |  |  |
| Physician | 2679 | 1439 | 1247 | <0.001 |
|  | N = 616 | N = 607 | (190) |  |
| Physician Assistant | 1586 | 1164 | 421 | 0.195 |
|  | N = 98 | N = 100 | (325) |  |
| Advanced Practice Nurse | 1897 | 945 | 946 | 0.001 |
|  | N = 205 | N = 200 | (289) |  |
| Nurse | 1645 | 886 | 762 | 0.003 |
|  | N = 296 | N = 310 | (255) |  |
| Assistant | 844 | 943 | -55 | 0.886 |
|  | N = 48 | N = 47 | (384) |  |

Mean dollars reported for gain and loss designs with sample size. Difference (gain – loss) is the incremental difference from linear regression with full set of interactions between randomized gain vs. loss designs and rurality (Model 2), experience with incentives (Model 3), and training (Model 4). All models also control for clinic specialty, number of clinics in healthcare system, practice type, number of providers in the clinic, percent of children covered by Vaccines for Children, number of children aged 9-12 years in typical week, gender, race/ethnicity, and years of experience. Robust standard errors of the gain – loss difference reported. p-values for tests of the null hypothesis that means for gain and loss are equal.
