# Supplementary material for: Gains vs losses in pay-for-performance: Stated preference evidence from a U.S. survey
Source: PLoS One. 2025 Feb 10;20(2):e0318704. doi: 10.1371/journal.pone.0318704 (PMC11809869; doi:10.1371/journal.pone.0318704)
Supplement: S2 Table — (DOCX) [file pone.0318704.s002.docx]

**S2 Table. Incentive Needed to Change Behavior ($): Gain vs. Loss Dropping Respondents with Zero Children Seen**

|  | Gain | Loss | Difference: Gain – Loss | p-value |
| --- | --- | --- | --- | --- |
| **Model 1: Main effect** |  |  |  |  |
| Full sample | 2153 | 1192 | 961 | <0.001 |
|  | N = 1251 | N = 1243 | (125) |  |
| **Model 2: Rural stratification** |  |  |  |  |
| Rural | 2375 | 1155 | 1220 | 0.013 |
|  | N = 116 | N = 110 | (493) |  |
| Non-rural | 2131 | 1195 | 935 |  |
|  | N = 1135 | N = 1133 | (128) | <0.001 |
| **Model 3: Experience stratification** |  |  |  |  |
| Experience with incentives | 2319 | 1218 | 1102 | <0.001 |
|  | N = 396 | N = 353 | (225) |  |
| No experience with incentives | 2076 | 1182 | 895 | <0.001 |
|  | N = 855 | N = 890 | (150) |  |
| **Model 4: Training stratification** |  |  |  |  |
| Physician | 2698 | 1448 | 1250 | <0.001 |
|  | N = 609 | N = 597 | (192) |  |
| Physician Assistant | 1586 | 1164 | 422 | 0.197 |
|  | N = 98 | N = 100 | (328) |  |
| Advanced Practice Nurse | 1903 | 942 | 961 | 0.001 |
|  | N = 204 | N = 195 | (293) |  |
| Nurse | 1596 | 893 | 708 | 0.005 |
|  | N = 292 | N = 305 | (250) |  |
| Assistant | 844 | 962 | -118 | 0.755 |
|  | N = 48 | N = 46 | (377) |  |

Mean dollars reported for gain and loss designs with sample size. Difference (gain – loss) is the incremental difference from linear regression with full set of interactions between randomized gain vs. loss designs and rurality (Model 2), experience with incentives (Model 3), and training (Model 4). This analysis dropped 33 respondents who reported seeing zero children aged 9-12 years in a typical week. Robust standard errors of the gain – loss difference reported. p-values for tests of the null hypothesis that means for gain and loss are equal.
