# Supplementary material for: Gains vs losses in pay-for-performance: Stated preference evidence from a U.S. survey
Source: PLoS One. 2025 Feb 10;20(2):e0318704. doi: 10.1371/journal.pone.0318704 (PMC11809869; doi:10.1371/journal.pone.0318704)
Supplement: Attachment — Submitted filename: Response to Reviewers v2.docx [file pone.0318704.s006.docx]

*We thank the reviewers again for their thoughtful comments and suggestions. Below we address each comment (in italics) and note where in the manuscript changes were made.*

**ACADEMIC EDITOR:**

1. We can see that in the main text, some references changed numbers, but in the reference section, this change is not mentioned through tracking.

*We have tracked changes that were made to the References, in text and in the bibliography, between the original submission and the revised document.*

2. We would be pleased if you revised the manuscript, taking into account the first referee's evaluation.

*In the revised manuscript, we removed all references to “framing” and focused on the difference between designing P4P incentives as gains vs losses.*

**Reviewer #1:** The revision was responsive and well done. With one exception, and that might be my fault. I explained on the first review that this study did not test gain v loss framing. The authors responded by dropping reference to prospect theory. But I think it is still a mistake to call this gain vs loss framing. That language is more appropriate when we are comparing to identical situations but merely framing them differently. You like a surgery with a 90% survival rate better than one with a 10% mortality rate, even those are two ways of framing the same thing.

In this study, the gain v loss conditions are not the same intervention with a different framing. They are different interventions. Better language would be something that emphasizes the relative impact of carrots vs sticks, of gains vs losses; but you should emphasize, so readers don’t make the same mistake, that you did not test gain v loss framing. If that isn’t clear, I’m happy to elaborate.

*Thank you for your clarification. In the revised manuscript, we removed all references to “framing” and focused on the difference between designing P4P incentives as gains vs losses.*

**Reviewer #2:**

1. We have updated citations to cite the underlying authors rather than websites.

Response to 1: I apologize but I cannot see where these changes have taken place because there are no tracked changes concerning the reference list. I can see that in the main text, some references changed numbers, but cannot verify whether citations now focus on authors rather than websites. I trust this is the case.

*We have tracked changes that were made to the References, in text and in the bibliography, between the original submission and the revised document.*
